# Structure and function of type IV IRES in picornaviruses: a systematic review

**DOI:** 10.3389/fmicb.2024.1415698

**Published:** 2024-05-24

**Authors:** Yan Li, Lei Zhang, Ling Wang, Jing Li, Yanwei Zhao, Fuxiao Liu, Qianqian Wang

**Affiliations:** ^1^College of Veterinary Medicine, Qingdao Agricultural University, Qingdao, China; ^2^Qingdao Center for Animal Disease Control and Prevention, Qingdao, China; ^3^Shandong New Hope Liuhe Group Co., Ltd., Qingdao, China; ^4^University Hospital, Qingdao Agricultural University, Qingdao, China; ^5^Market Supervision Administration of Huangdao District, Qingdao, China

**Keywords:** picornavirus, IRES, type IV IRES, stem-loop structure, ribosome, host factor, translation

## Abstract

The *Picornaviridae* is a family of icosahedral viruses with single-stranded, highly diverse positive-sense RNA genomes. Virions consist of a capsid, without envelope, surrounding a core of RNA genome. A typical genome of picornavirus harbors a well-conserved and highly structured RNA element known as the internal ribosome entry site (IRES), functionally essential for viral replication and protein translation. Based on differences in their structures and mechanisms of action, picornaviral IRESs have been categorized into five types: type I, II, III, IV, and V. Compared with the type IV IRES, the others not only are structurally complicated, but also involve multiple initiation factors for triggering protein translation. The type IV IRES, often referred to as hepatitis C virus (HCV)-like IRES due to its structural resemblance to the HCV IRES, exhibits a simpler and more compact structure than those of the other four. The increasing identification of picornaviruses with the type IV IRES suggests that this IRES type seems to reveal strong retention and adaptation in terms of viral evolution. Here, we systematically reviewed structural features and biological functions of the type IV IRES in picornaviruses. A comprehensive understanding of the roles of type IV IRESs will contribute to elucidating the replication mechanism and pathogenesis of picornaviruses.

## 1 Introduction

The family *Picornaviridae* comprises a large group of RNA viruses, including several significant human and animal pathogens (Zell, 2018). Since the discovery of the first picornavirus, foot-and mouth disease virus in 1898, this family has expanded to include at least 158 recognized species divided into 68 genera (Ng et al., 2015; Zell et al., 2021). These viruses have single positive-stranded RNA genomes ranging from 6.7 to 10.1 kilonucleotides in length (Andino et al., 2023). Although picornaviruses express different proteins, their RNA genomes share a similar organization (Yang et al., 2002). The picornaviral genome codes for a single polyprotein, subsequently cleaved into multiple mature structural and nonstructural proteins by virus-encoded proteinases (Zell, 2018). The 5′ end of picornaviral genome lacks a cap structure present in eukaryotic mRNAs. Instead, it is covalently linked to a virus-encoded protein, VPg, serving as a primer for RNA synthesis (Martinez-Salas et al., 2015). Immediately following VPg, there is a long and highly structured 5′ untranslated region (5’ UTR) containing the internal ribosome entry site (IRES), a *cis*-acting element essential for the synthesis of viral polyprotein (Daijogo and Semler, 2011).

The IRES element is an RNA fragment capable of folding into a complex structure, enabling itself to interact with one or more components of the canonical translation apparatus in a specific, cap-independent manner (Fernández-Miragall and Martínez-Salas, 2007; Firth and Brierley, 2012; Holmes and Semler, 2019). These interactions facilitate the recruitment of ribosomes or ribosomal pre-initiation complexes to an internal site in mRNA following infection with RNA viruses. By bypassing the conventional process of translation initiation, IRES allows for the continuous synthesis of viral proteins, even in the presence of the host’s translational shutdown of its own proteins (Jackson et al., 2010; Yu et al., 2011b; Firth and Brierley, 2012; Arhab et al., 2020). Consequently, RNA viruses can effectively evade antiviral defenses in hosts.

Due to their diversities in structure and function, picornaviral IRES elements have been classified into five distinct types (Martinez-Salas et al., 2017). The type IV IRES is specifically characterized by its compact structure and minimal reliance on eukaryotic initiation factors (eIFs) (Sweeney et al., 2014). Moreover, the wide distribution of type IV IRESs across numerous genera in the *Picornaviridae* family (Arhab et al., 2020) has aroused widespread interest among virologists. Here, we systematically reviewed the type IV IRES, mainly involved in its structure, function and impact on the initiation of polyprotein translation.

## 2 Classification of picornaviral IRESs

IRESs were originally found in picornaviruses, such as poliovirus (PV) (Pelletier and Sonenberg, 1988) and encephalomyocarditis virus (EMCV) (Jang et al., 1988). Subsequently, the presence of IRESs has been identified in many other viruses and cellular mRNAs (Yang and Wang, 2019). In recent years, with in-depth analysis on their structure- and action-related mechanisms, picornavirus IRESs have been classified into five types, designated type I, II, III, IV, and V. The type IV and V are also referred to as hepatitis C virus (HCV)-like IRES and aichivirus (AV)-like IRES, respectively (Sweeney et al., 2014). Each type of IRES element contains specific RNA secondary structures that vary across IRES types. These structures can exclusively recognize and bind specific factors, such as the 40S ribosomal subunit, a subset of eIFs, and several RNA-binding proteins (RBPs). Through these specific interactions, one IRES is able to recruit directly the ribosome to a viral start codon, facilitating the pathway of translation initiation, distinct from that of the cap-binding initiation mode (Lozano and Martínez-Salas, 2015).

**Figure 1:**
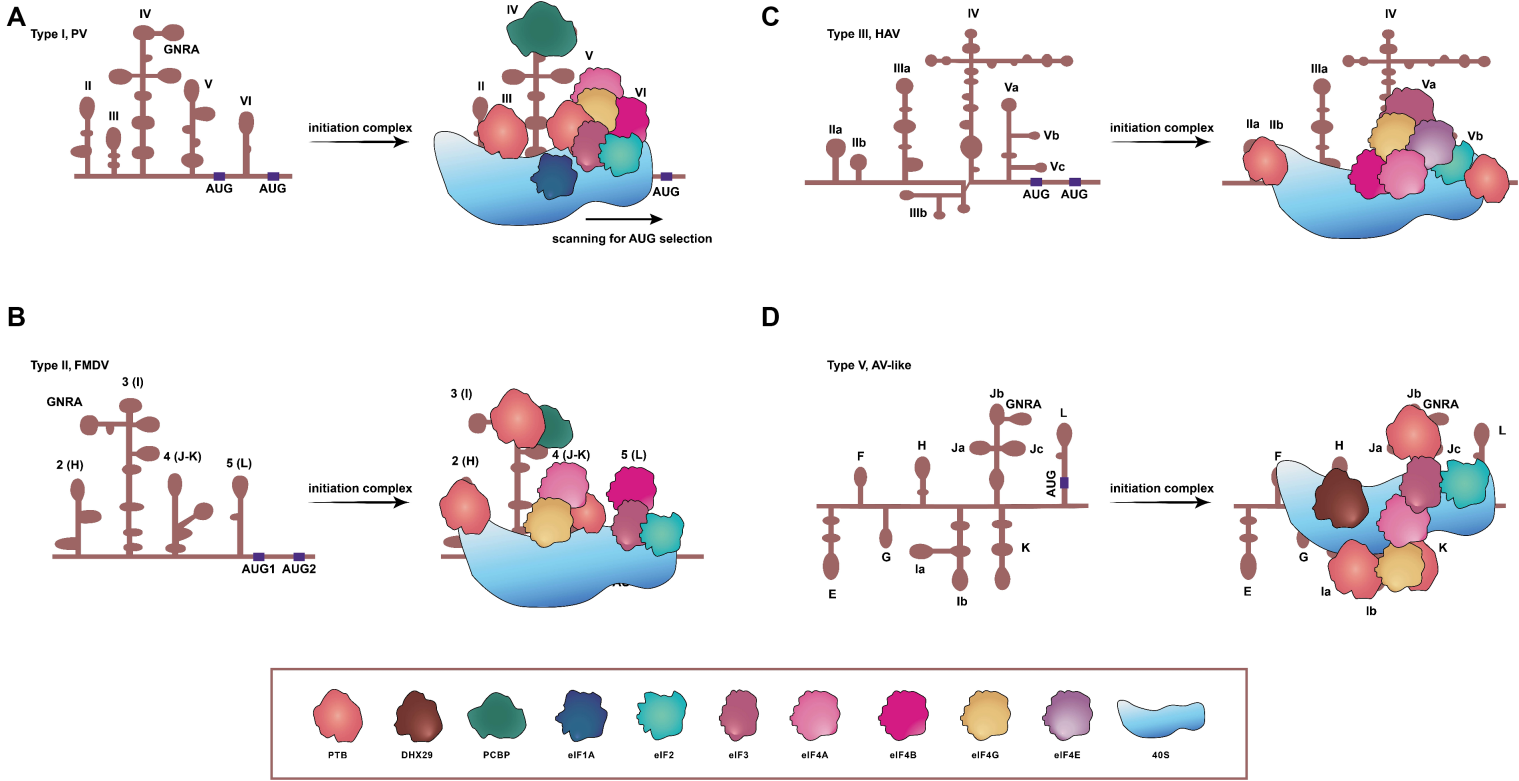
Schematic representations of secondary structures, and requirements of translation initiation factors for four IRESs in picornaviruses. **(A)** Type I IRES, exemplified by PV, comprises five principal structural domains (dII to dVI). It involves all translation initiation factors and many ITAFs to initiate translation, concerning a scanning process. **(B)** FMDV contains the type II IRES, arranged in modular domains (H to L). The type II IRES does not scan the mRNA, and instead, the 48S-like complex is recruited directly to the start codon. **(C)** The type III IRES has been found only in HAV, and the protein translation mediated by this type depends on eIF4E. **(D)** The type V IRES is present in AV and harbors eight domains. Distinct from all previously characterized IRESs, the one of AV exhibits an absolute dependency on DHX29, necessitated by the entrapment of its AUG within a stable hairpin structure. DHX29: DExH-box protein DHX29; eIF1A, eIF2, eIF3, eIF4A, eIF4B, eIF4E, and eIF4G: eukaryotic initiation factor 1A, 2, 3, 4A, 4B, 4E, and 4G. PTB: pyrimidine tract-binding protein; PCBP: poly(C)-binding protein; 40S: 40S small ribosomal subunit.

Type I, II, and III IRESs, exemplified by PV, foot-and-mouth disease virus (FMDV) and hepatitis A virus (HAV), respectively, utilize nearly all canonical translation initiation factors, as well as non-canonical ones known as IRES *trans*-acting factors (ITAFs) (Pacheco and Martinez-Salas, 2010), to enhance the IRES activity (Hanson et al., 2012). Although initiation factors, like eIF4E and intact eIF4G, are superfluous for type I and II IRESs, these factors are essential for the function of type III IRESs (Ali et al., 2001; Sadahiro et al., 2018). Similarly, the type V IRES found in AV involves multiple canonical eIFs during its process of translation initiation (Yu et al., 2011b; Sweeney et al., 2012). Distinct from other IRES categories, type IV elements demonstrate a diminished dependence on eIFs and eliminate the requirement for ITAFs in assembling 48S complexes (Pisarev et al., 2004; Mailliot and Martin, 2018). These IRES-mediated translation mechanisms are primarily associated with two models (Komar and Hatzoglou, 2015): the first one, whereby the 40S ribosome positions itself in the vicinity of AUG, subsequently scanning to locate the AUG (Figure 1A), and the second one, in which the 40S ribosomal subunit directly localizes to the AUG (Figures 1B–D).

The type I IRES in picornaviruses is composed of five principal domains, designated dII to dVI (Martinez-Salas et al., 2015). Translation initiation on PV IRES entails the scanning by 43S ribosomal preinitiation complexes, and engages a set of eIFs (eIF2, eIF3, eIF4A, eIF4G, eIF4B, and eIF1A) and a single ITAF, identified as the poly(C)-binding protein 2 (PCBP2), as shown in Figure 1A (Sweeney et al., 2014; Andreev et al., 2022). In instance of conventional pathways of translation initiation inaccessible, these viruses can employ an alternative IRES-independent transition mechanism, which depends on eIF2A/eIF2D and utilizes a non-AUG codon for initiation. This alternative pathway seems to facilitate ongoing translation and viral genome replication in the presence of activated intrinsic defenses against viruses (Kim et al., 2023).

Similar to type I IRESs, type II IRESs, such as those of EMCV and FMDV, also comprise five major domains, designated H to L (Yu et al., 2011a). Translation initiation mediated by IRESs of EMCV and FMDV involves the specific binding of eIF4G and eIF4A to the Y-shaped J-K domain, an interaction dependent on a conserved sequence/structural motif at the apex of domain J (Figure 1B) (López de Quinto and Martínez-Salas, 2000; Yu et al., 2011a; Sweeney et al., 2012; Imai et al., 2016). In EMCV, the IRES structure can be remodeled by eIF4G/eIF4A to promote EMCV-IRES/40S interactions, and rearrange the coding region to accommodate correctly the AUG in the ribosomal mRNA cleft. The 43S complex can then undergo structural rearrangement due to both AUG/tRNA recognition and close contact between the ribosome and IRES. This would allow the 60S to join the complex and to initiate translation (Chamond et al., 2014). Type II IRESs therefore can function dependent neither on the eIF4E nor on factors implicated in ribosomal scanning, such as eIF1 and eIF1A (Sweeney et al., 2012).

The secondary structure of the HAV IRES element comprises six structural domains, and this IRES element is much longer than types I and II (Brown et al., 1991; Francisco-Velilla et al., 2022). HAV IRES requires the intact eIF4G to initiate translation, unlike types I and II, both of which require only the C-terminal two-thirds fragment of eIF4G (Francisco-Velilla et al., 2022). The absence of a cap structure at the 5′ end of picornaviral genome indicates that the involvement of the cap-binding initiation factor eIF4E is unnecessary for the translation initiation of picornaviruses. However, eIF4E is essential in HAV for the activation of IRES-mediated translation (Figure 1C) (Redondo et al., 2012). Thus, HAV is unable to shut down the protein synthesis in hosts by a similar mechanism as those of other picornaviruses, and its IRES is inefficient, probably due to its unfair competition for the cellular translation machinery and tRNAs (Pinto et al., 2018, 2021). To ameliorate the tRNA competition, HAV has evolved a highly biased and deoptimized codon usage with respect to its hosts (Pinto et al., 2007; Costafreda et al., 2014; Pinto et al., 2018). During translation, the eIF4F complex recognizes the IRES element and, subsequently, the 43S preinitiation complex and the 60S large ribosomal subunits are directly recruited onto HAV mRNA to synthesize the viral polyprotein (Sadahiro et al., 2018).

The AV IRES consists of eight domains, designated E to L. *In vitro* reconstitution of initiation on the AV IRES reveals that it shares some characteristics with type I and II IRESs, suggesting that it appears to be a chimera between type I and type II (Yu et al., 2011b; Sweeney et al., 2012). The formation of 48S complex on the AV IRES requires eIF2, eIF3, eIF4A, the eIF4A-interacting central domain of eIF4G (eIF4Gm) and the DExH-box protein DHX29. This process is strongly stimulated by the pyrimidine tract-binding protein (Figure 1D). The lack of dependence on eIF1 and eIF1A, which robustly stimulate scanning and monitor the fidelity of AUG selection during this process, implies that the AUG can be directly recognized and bound by the 43S complex on the AV IRES, hence bypassing the need for scanning (Yu et al., 2011b).

**Figure 2:**
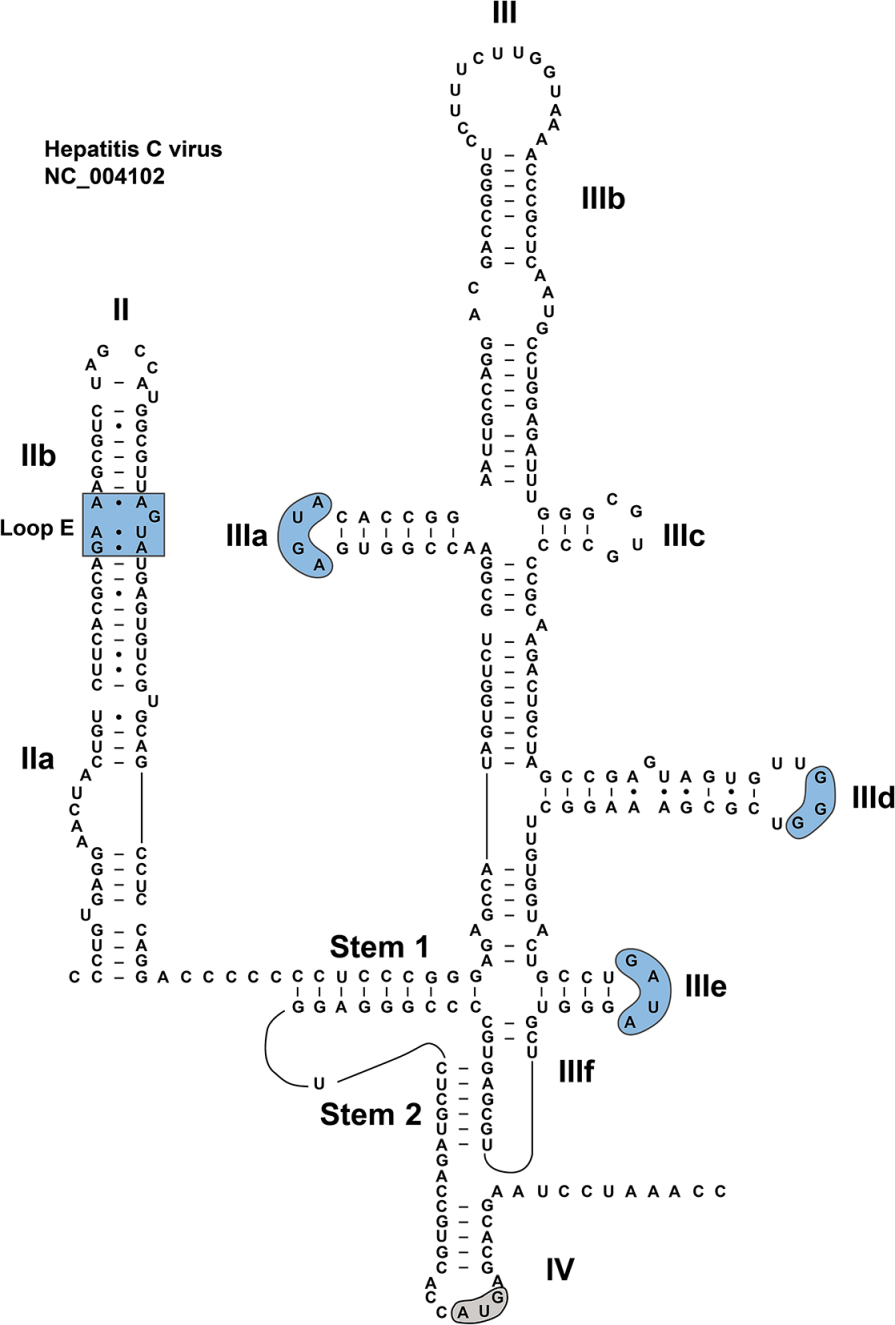
RNA sequence and secondary structure of HCV IRES. The conserved motifs in dII and dII are indicated by blue shades. The start codon is indicated by a gray shade.

The translation initiation mediated by the type IV IRES occurs through direct binding of the IRES element to the 40S ribosomal subunit. The IRES–40S complex positions the AUG at the P site and forms a 48S complex with eIF2/GTP/initiator tRNA (Hashem et al., 2013), stabilized by the binding of eIF3 (Ji et al., 2004). Following two steps of GTP hydrolysis, eIF2 is released, and the 60S ribosomal subunit associates under the action of eIF5B, resulting in the formation of an active 80S ribosome. This 80S ribosome subsequently initiates the synthesis of viral polyprotein (Figure 2) (Ji et al., 2004; Johnson et al., 2017; Niepmann and Gerresheim, 2020).

The relationship between RNA structure and biological function of distinct IRESs has been intensively analyzed by various experimental methods (Malnou et al., 2002; Bailey and Tapprich, 2007; Serrano et al., 2009), providing insights into the initiation mechanism of viral protein translation. Since the secondary and tertiary structures of type IV IRES are very different from those of the other four types, the type IV IRES is systematically reviewed here.

## 3 Identification of type IV IRES in picornaviruses

The type IV IRES was initially identified in HCV, bovine viral diarrhea virus (BVDV) and classical swine fever virus (CSFV) (Arhab et al., 2020). This specific IRES type is subsequently found in approximately 25 distinct genera in the *Picornaviridae* family (Table 1) (Arhab et al., 2020). In the context of the *Picornaviridae* family, the porcine teschovirus-1 (PTV), belonging to the *Teschovirus* genus, stands as one of the earliest documented instances (Pisarev et al., 2004). Although the major portion of the PTV-1 genome had been determined prior to 2002, its 5′-terminal sequence had been neither cloned nor characterized (Kaku et al., 2002). Kaku and colleagues subsequently demonstrated the presence of a functional IRES element in the PTV 5’ UTR by constructing and analyzing plasmids that expressed bicistronic mRNAs. Further tests confirmed that the activity of this element was unaffected by the co-expression of an enterovirus 2A protease and the cleavage of eIF4G. It could also function effectively in an RRL (rabbit reticulocyte lysate) *in vitro* translation system (Kaku et al., 2002). Thus, the biological properties of the PTV IRES are most similar to those of EMCV and FMDV IRES elements, but the computer-predicted secondary structure of the PTV IRES shows no apparent resemblance to those of cardiovirus and aphthovirus IRES elements. The PTV IRES is consequently classified into a new category of IRES (Kaku et al., 2002).

**Table 1:** Current classification of picornaviruses with type IV IRES.

Genus	Species	Identified viruses	GenBank No.	Genome size (nt)	IRES size (nt)	Loop E motif	“8”-like structure	Structural domains	References
*Aalivirus*	*Aalivirus A*	Aalivirus A1	KJ000696.1	8,976	267	+	+	II, III	Wang et al. (2014)
*Anativirus*	*Anativirus A*	Avian anativirus 1	AY563023.1	8,289	316	+	−	II, III	Tseng and Tsai (2007)
*Aquamavirus*	*Aquamavirus A*	Seal picornavirus 1	EU142040.1	6,718	196	−	+	II, III	Kapoor et al. (2008)
*Aquamavirus*	*Aquamavirus B*	Bear picornavirus 1	MH760796.1	6,703	/	/	+	/	Wang et al. (2019b)
*Avihepatovirus*	*Avihepatovirus A*	Duck hepatitis virus 1	DQ249299.1	7,687	262	+	+	II, III	Hellen and de Breyne (2007), Tseng et al. (2007)
*Colbovirus*	Unassigned	Pigeon picornavirus B	KC560801.3	7,971	417	+	+	II, III, IV	Kofstad and Jonassen (2011), Asnani et al. (2015)
*Crohivirus*	*Crohivirus B*	Bat crohivirus	NC_033819.1	7,085	334	+	−	II, III, IV	Yinda et al. (2017), Arhab et al. (2020)
*Diresapivirus*	*Diresapivirus A*	Diresapivirus A1	KJ641685.1	6,624	/	−	−	II, III	Wu et al. (2016)
*Diresapivirus*	*Diresapivirus B*	Diresapivirus B1	KJ641697.1	7,048	251	−	−	II, III	Wu et al. (2016)
*Felipivirus*	*Felipivirus A*	Feline picornavirus	JN572117.1	7,415	239	+	−	II, III	Lau et al. (2012), Asnani et al. (2015)
*Grusopivirus*	*Grusopivirus A*	Grusopivirus A1	KY312544.1	7,917	266	+	−	II, III	Wang Y. et al. (2019)
*Grusopivirus*	*Grusopivirus C*	Lorikeet picornavirus 1	MK443503.1	7,862	/	/	+	/	Wang et al. (2019a)
*Hepatovirus*	*Hepatovirus C*	Bat hepatovirus	NC_038313.1	7,570	322	−	−	II, III, IV	Drexler et al. (2015), Arhab et al. (2020)
*Kobuvirus*	*Aichivirus B*	Ferret kobuvirus	KF006985.1	8,052	297	+	−	II, III	Asnani et al. (2015)
*Kobuvirus*	*Aichivirus C*	Porcine kobuvirus 1	NC_011829.1	8,210	293	+	−	II, III	Reuter et al. (2009)
*Kunsagivirus*	*Kunsagivirus A*	Kunsagivirus A1	KC935379.1	7,272	189	−	−	II, III	Boros et al. (2013), Asnani et al. (2015)
*Kunsagivirus*	*Kunsagivirus B*	Bat kunsagivirus	KX644936.1	7,092	179	−	−	II, III	Asnani et al. (2015), Yinda et al. (2017)
*Kunsagivirus*	*Kunsagivirus C*	Kunsagivirus C1	KY670597.1	7,429	/	/	/	/	Buechler et al. (2017)
*Limnipivirus*	*Limnipivirus A*	Bluegill picornavirus	JX134222	8,050	251	−	−	II, III, IV	Barbknecht et al. (2014), Asnani et al. (2015)
*Limnipivirus*	*Limnipivirus B*	Carp picornavirus 1	KF306267	7,697	233	−	−	II, III, IV	Lange et al. (2014), Asnani et al. (2015)
*Limnipivirus*	*Limnipivirus C*	Fathead minnow picornavirus	KC465953.1	7,934	237	−	−	II, III, IV	Phelps et al. (2014), Asnani et al. (2015)
*Ludopivirus*	*Ludopivirus A*	Goose picornavirus 1	MF358731.1	8,051	299	−	−	II, III	Boros et al. (2018)
*Megrivirus*	*Megrivirus A*	Turkey hepatitis virus 1	HM751199.1	9,075	397	+	+	II, III	Honkavuori et al. (2011)
*Megrivirus*	*Megrivirus A*	Duck megrivirus	KC663628.1	9,700	398	+	+	II, III	Liao et al. (2014)
*Megrivirus*	*Megrivirus A*	Goose megrivirus	KY369299.1	9,840	/	/	/	/	Wang et al. (2017)
*Megrivirus*	*Megrivirus B*	Pigeon mesiviruses	KC876003.1	9,101	423	+	+	II, III, IV	Phan et al. (2013), Asnani et al. (2015)
*Megrivirus*	*Megrivirus C*	Chicken megrivirus	KF961186.1	9,560	396	+	+	II, III	Boros et al. (2014a), Lau et al. (2014), Kim et al. (2015), Haji Zamani et al. (2023)
*Megrivirus*	*Megrivirus E*	Penguin megrivirus	MF405436.1	9,702	/	/	/	/	Zell (2018), Yinda et al. (2019)
*Mosavirus*	*Mosavirus B*	Marmot mosavirus	KY855435.1	8,170	259	−	−	II, III	Luo et al. (2018), Arhab et al. (2020)
*Parechovirus*	*Parechovirus D*	Ferret parechovirus	KF006989.1	7,066	329	+	−	II, III	Smits et al. (2013)
*Parechovirus*	Unassigned	Manhattan parechovirus	KJ950935.1	/	316	+	−	II, III	Firth et al. (2014)
*Pasivirus*	*Pasivirus A*	Swine pasivirus 1	JQ316470.1	6,916	286	−	−	II, III, IV	Sauvage et al. (2012), Yu et al. (2013), Asnani et al. (2015)
*Pemapivirus*	*Pemapivirus A*	Pemapivirus A1	MG600106.1	9,196	260	−	−	II, III	Arhab et al. (2020)
*Phacovirus*	Unassigned	Quail picornavirus 1	JN674502.1	8,159	345	−	+	II, III	Pankovics et al. (2012)
*Rafivirus*	*Rafivirus A*	Tortoise rafivirus A1	KJ415177.1	7,204	264	+	−	II, III	Asnani et al. (2015), Ng et al. (2015)
*Sakobuvirus*	*Sakobuvirus A*	Feline sakobuvirus A	KF387721.1	7,807	274	+	−	II, III	Ng et al. (2014)
*Sapelovirus*	*Sapelovirus A*	Porcine sapelovirus 1	KJ821020.1	7,566	277	−	−	II, III	Krumbholz et al. (2002), Son et al. (2014)
*Sapelovirus*	*Sapelovirus B*	Simian sapelovirus	NC_004451.1	8,126	367	+	−	II, III, IV	Oberste et al. (2003), Hellen and de Breyne (2007)
*Sapelovirus*	Unassigned	California sea lion sapelovirus 1	JN420370.2	7,497	269	−	−	II, III	Li et al. (2011)
*Senecavirus*	*Senecavirus A*	Senecavirus A	DQ641257.1	7,310	304	−	−	II, III	Willcocks et al. (2011)
*Symapivirus*	*Symapivirus A*	Symapivirus A1	MG600076.1	8,591	254	−	−	II, III	Phelps et al. (2014), Asnani et al. (2015)
*Teschovirus*	*Teschovirus A*	Porcine teschovirus	AB038528.1	7,088	219	−	−	II, III	Pisarev et al. (2004), Hellen and de Breyne (2007)
*Tremovirus*	*Tremovirus A*	Avian encephalomyelitis virus	AJ225173.1	7,055	294	+	−	II, III	Hellen and de Breyne (2007), Bakhshesh et al. (2008)
*Tropivirus*	*Tropivirus A*	Tropivirus A1	MG600091.1	8,049	346	+	−	II, III	Arhab et al. (2020)
Unassigned	Unassigned	*Ia io* picornavirus 1	JQ814852.1	7,543	239	−	−	II, III	Wu et al. (2016)
Unassigned	Unassigned	Bat picornavirus 1	HQ595340.1	7,753	276	−	−	II, III	Lau et al. (2011)
Unassigned	Unassigned	Bat picornavirus 2	HQ595342.1	7,963	/	−	−	II, III	Lau et al. (2011)
Unassigned	Unassigned	Guanxi changeable lizard picornavirus 2	MG600105.1	7,115	247	+	−	II, III	Arhab et al. (2020)
Unassigned	Unassigned	Pink-eared duck picornavirus	MK204421.1	6,696	217	−	−	II, III	Arhab et al. (2020)

+ Presence; − Absence; / Undefined.

Following the definition of the type IV IRES, this group further determined its boundaries and identified the requirements for the formation of 48S preinitiation complexes through toeprinting assays. It was found that the formation of 48S preinitiation complex on the PTV-1 IRES had no need of the initiation factors eIF1, eIF1A, eIF3, eIF4B, and eIF4F (Pisarev et al., 2004). It only involves purified 40S ribosomal subunits plus the ternary complex of eIF2, Met-tRNA, and GTP, although this process is enhanced in the presence of eIF3. Indeed, the PTV-1 IRES can form a binary complex with 40S subunits alone (Chard et al., 2006a,b). These data demonstrate that the PTV-1 IRES has properties completely different from those of other IRES elements in picornaviruses, but highly resembles to the HCV IRES (Pisarev et al., 2004). Therefore, this unique class of type IV IRES is also referred to as HCV-like IRES.

Interestingly, the “unique” IRES was subsequently found in the *Sapelovirus* (Krumbholz et al., 2002; Oberste et al., 2003; Li et al., 2011; Son et al., 2014) and *Anativirus* genera (Tseng and Tsai, 2007). Functional assays demonstrated that these IRES elements remained still active, when eIF4G was cleaved and when the activity of eIF4A was blocked (Chard et al., 2006a). On the basis of the sequence information and the structure prediction, the type IV IRES has also been demonstrated to exist in the *Kobuvirus* (Reuter et al., 2009), *Aalivirus* (Wang et al., 2014), *Tremovirus* (Hellen and de Breyne, 2007; Bakhshesh et al., 2008), *Avihepatovirus* (Tseng et al., 2007; Pan et al., 2012), *Pasivirus* (Sauvage et al., 2012; Yu et al., 2013; Asnani et al., 2015), and *Senecavirus* genera (Willcocks et al., 2011).

In addition to the aforementioned picornaviruses that mainly infect pigs and poultry, the others harboring the type IV IRES have also been found in a wide range of vertebrates from fishes to mammals. For example, seal picornavirus type 1 (SePV-1) was first identified in marine mammals in 2007, and then classified into the genus *Aquamavirus* (Kapoor et al., 2008). The second member of this genus is bear picornavirus 1 (BePV-1), another novel picornavirus isolated from black bear. Sequence analyses of SePV-1 and BePV-1 revealed that both of them had the type IV IRES (Wang et al., 2019b). In 2011, three novel picornaviruses, bat picornavirus 1 (BPV-1), BPV-2, and BPV-3, were identified in bats. Both BPV-1 and BPV-2 were characterized by the presence of the type IV IRES, whereas the BPV-3 harbored the type I IRES (Lau et al., 2011; Zell, 2018). More picornaviruses have been recently identified in bat species, including *Hepatovirus* (Drexler et al., 2015), *Diresapivirus* (Wu et al., 2016), *Crohivirus* and *Kunsagivirus* (Asnani et al., 2015; Yinda et al., 2017) genera, all of which are found to possess the type IV IRES.

A novel feline picornavirus, FePV in the genus *Felipivirus*, was identified in stray cats in Hong Kong. FePV is closely related both to members in the genus *Sapelovirus* and to the unclassified BPV-3, but with the difference that FePV possesses the type IV IRES instead of the type I IRES found in BPV-3 (Lau et al., 2012). Feline sakobuvirus A, the second picornavirus with the type IV IRES isolated from cats, does not belong to the same genus as that of FePV, but exhibits a closer relation to members of the genus *Kobuvirus* (Ng et al., 2014). Type IV-containing picornaviruses are also recognized in other mammals, including rats (genus *Parechovirus*) (Firth et al., 2014), ferret (genus *Parechovirus*) (Smits et al., 2013), marmot (genus *Mosavirus*) (Luo et al., 2018) and baboons (genus *Kunsagivirus*) (Buechler et al., 2017).

The genus *Megrivirus*, recognized to contain one of the longest picornaviral genomes currently described, comprises five species, named A to E. The type IV IRES is present in the 5’ UTR region of all species, with the sole exception of species D (Honkavuori et al., 2011; Phan et al., 2013; Lau et al., 2014; Liao et al., 2014; Boros et al., 2014a; Kim et al., 2015; Wang et al., 2017; Zell, 2018; Yinda et al., 2019; Haji Zamani et al., 2023). This type of virus is additionally found in migrant bird (genus *Kunsagivirus*) (Boros et al., 2013), pigeon (genus *Colbovirus*) (Kofstad and Jonassen, 2011), quail (genus *Phacovirus*) (Pankovics et al., 2012), goose (genus *Ludopivirus*) (Boros et al., 2018), duck (Pink-eared duck picornavirus), red-crowned crane (genus *Grusopivirus*) (Wang Y. et al., 2019; Arhab et al., 2020), lorikeet (genus *Grusopivirus*) (Wang et al., 2019a), as well as other species, such as amphibians, reptiles (Ng et al., 2015; Arhab et al., 2020) and fishes (Table 1) (Barbknecht et al., 2014; Lange et al., 2014; Phelps et al., 2014; Asnani et al., 2015). Most known type IV-containing picornaviruses are found in mammals and birds, whereas this type of picornavirus has also been identified in other vertebrates in recent years. These findings further emphasize the relative ubiquity and diversity of the type IV IRES.

## 4 Structure elements of type IV IRES in picornaviruses

**Figure 3:**
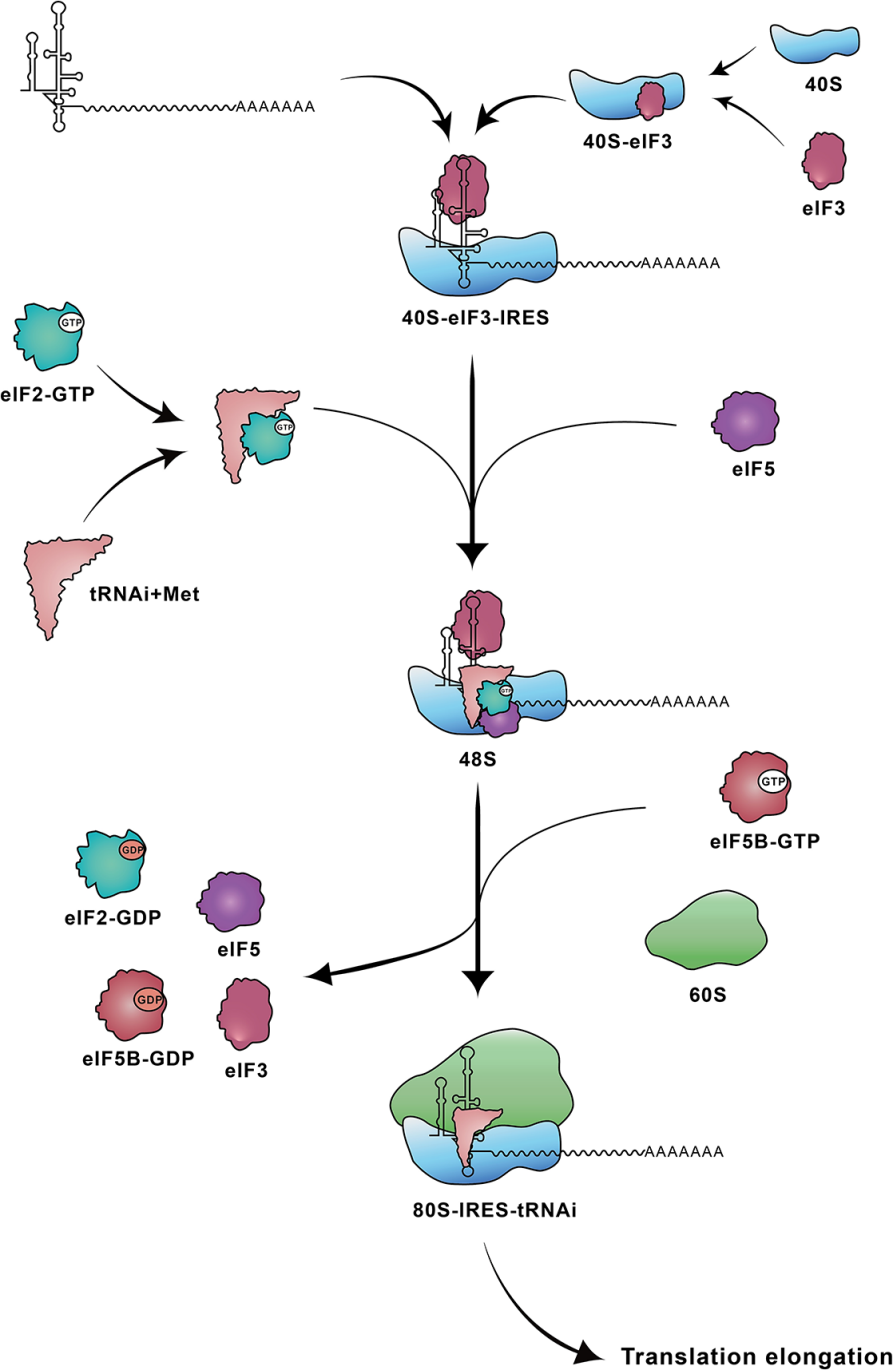
Steps involved in translation initiation mediated by HCV IRES. The HCV IRES first binds to a 40S-eIF3 complex. Subsequently the ternary 40S-IRES-eIF3 complex acquires the eIF2-tRNAi^Met^-GTP and eIF5. Following two steps of GTP hydrolysis, the 60S ribosomal subunit joins to form the elongation-competent 80S ribosome. 40S: 40S small ribosomal subunit; 40S-eIF3: 40S ribosomal subunit-eukaryotic initiation factor 3; 48S: 48S complex; 60S: 60S large ribosomal subunit; 80S-IRES-tRNAi: 80S-IRES-initiator tRNA; eIF2-GTP: eIF2-guanosine triphosphate; eIF2-GDP: eIF2-guanosine diphosphate; eIF5B-GTP: eIF5B-guanosine triphosphate; eIF5B-GDP: eIF5B-guanosine diphosphate; tRNAi+Met: tRNAi-methionine.

A great deal of works have been devoted to studies on the HCV IRES (Lukavsky, 2009; Berry et al., 2011; Perard et al., 2013; Yamamoto et al., 2015; Yokoyama et al., 2019; Brown et al., 2022), which is of critical importance for understanding the biological mechanisms of the type IV IRES. The structure of HCV IRES can serve as an ideal model for unveiling secondary structures of type IV IRESs, especially considering the similar roles these elements play in the synthesis of viral proteins within their individual viruses. Although a significant portion of the currently identified HCV-like IRES structures are based on the model of HCV IRES for research, each of them has its own specific structure and function. The arrangement of type IV IRES is relatively simple, mainly composed of secondary stem–loop structures and tertiary structures (Asnani et al., 2015; Lee et al., 2017). A typical HCV IRES is approximately 340 nt in length, containing many stem–loop structures, known as dII, dIII, and dIV, a pseudoknot (PK), and a helical structure that links dII with dIII and dIV (Kalliampakou et al., 2002). DII is a 70-nt-long flexible hairpin, composed of two subdomains, IIa and IIb: the former harboring an asymmetric internal loop; the latter including an internal loop E motif and an apical hairpin (Figure 3). These structural features are relatively conserved among closely related HCV-like IRESs in members of the *Flaviviridae* family (Locker et al., 2007; Lukavsky, 2009).

DIII consists of several subdomains (IIIa to IIIf). The basal part of dIII contains a 4-way junction, which includes a predicted PK (IIIf) and a small stem–loop (IIIe) (Melcher et al., 2003; Lukavsky, 2009). In the middle of the dIII, the dIIId forms part of a three-way junction. The apical region of dIII contains a 4-way junction (Figure 3), involved in a central dIII stem, as well as dIIIa, dIIIb and dIIIc (Koirala et al., 2020). Certain motifs in the HCV dIII also exhibit a high degree of conservation in some viruses, such as in CSFV and BVDV. For example, dIIIa and dIIIe contain AGUA and GA[U/C]A tetraloops, respectively. The dIIId features a G-rich loop, with at least three consecutive G residues, in the hairpin (Fletcher and Jackson, 2002; Lukavsky, 2009). However, the sequence of highly conserved dIIIa loop is unimportant for the maintenance of full IRES activity (Fletcher and Jackson, 2002). The dIV is the last subdomain in the type IV IRES, and harbors the viral start codon (Berry et al., 2011; Tanaka et al., 2018).

Over 40 structures associated with the type IV IRES have currently been identified or predicted. Representative models of type IV IRESs are schematically shown in Supplementary Figure S1. These IRESs are 180–420 nucleotides in length (Asnani et al., 2015), exhibiting the significant diversity in their structures, whereas all these IRES structures contain a crucial, highly conserved core region, including the PK as well as subdomains IIId, IIIe, and IIIf (Arhab et al., 2020). The core region is functionally essential for the translation initiation. The dIII is typically composed of four to seven subdomains in the type IV IRES, whereas the number of subdomains varies greatly among different picornaviruses. The simplest one is derived from Guangxi changeable lizard picornavirus 2, only having four subdomains: IIIa, IIId, IIIe, and IIIf. The most complex ones, like members in *Crohivirus*, *Parechovirus*, *Tropivirus*, *Senecavirus*, *Ludopivirus*, and *Tremovirus* genera, are composed of seven subdomains: IIIa to IIIf. In addition to the complete complement of characteristic subdomains and motifs, these complex IRESs also bear an extra dIIIa2 (*Ludopivirus* genus) (Boros et al., 2018), dIIId2 (*Senecavirus*, *Parechovirus*, *Crohivirus*, and *Tropivirus* genera) or dIIIb2 (*Tremovirus* genus) (Hellen and de Breyne, 2007; Arhab et al., 2020).

Several conserved sequences exist in dIII, including the apical GGG motif in dIIId and a GA[U/C]A tetraloop in dIIIe. An “AGUA” loop at the top of dIIIa, similar to that in HCV IRES, is only found in the genera *Pasivirus*, *Tremovirus*, *Tropivirus*, *Senecavirus*, *Crohivirus*, *Parechovirus*, and *Pasivirus* (Hellen and de Breyne, 2007; Asnani et al., 2015; Arhab et al., 2020). The GGG motif at the top of IIId loop was found to be a GGGGG pentaloop in a homologous position in simian sapelovirus (Hellen and de Breyne, 2007). In some cases, the G-rich sequence has four consecutive G residues in some picornaviruses, including in carp picornavirus 1, fathead minnow picornavirus, BPV-1, *Ia io* picornavirus 1, California sea lion sapelovirus 1, grusopivirus, FePV, tortoise rafivirus A1 and crohivirus B (Asnani et al., 2015; Arhab et al., 2020). However, only two G residues are identified in pink-eared duck picornavirus and pemapivirus (Arhab et al., 2020). The dIIIe, consisting of an apical GA[U/C]A tetraloop and a 4-bp stem, is conserved in almost all type IV IRESs, whereas members of the species *Megrivirus B* have a GAUC loop, and Aalivirus has a 3-bp stem and 5-nt apical loop (Wang et al., 2014; Asnani et al., 2015).

Significant sequence variations are observed in the apical region of dIII. For example, in *Kobuvirus*, *Kunsagivirus*, *Limnipivirus*, and *Rafivirus* genera, the apical region of dIII may be severely truncated, possibly lacking one (genera *Kunsagivirus*, *Limnipivirus*, *Sapelovirus*, and *Teschovirus*) or two (genus *Aalivirus*) subdomains (Arhab et al., 2020). Additionally, subdomains IIIa and IIIc may also be disposed in a staggered configuration rather than forming a 4-way junction. Subdomain IIIb may be greatly elongated, as observed in the *Megrivirus*, *Phacovirus*, and *Colbovirus* genera, which are topped by a 20-nt-long “8”-like structure (Asnani et al., 2015; Boros et al., 2016). Such a structure is conserved in avian-origin and seal picornaviruses, and may be crucial for the viral translation (Pankovics et al., 2012; Boros et al., 2014b).

The dII of type IV IRES, with a length range of 21–120 nt, is shorter or longer than that of the HCV IRES. Compared to the dIII, the dII shows the great variability. In *Teschovirus*, *Tropivirus*, and *Tremovirus* genera, the dII contains a small branching hairpin (dIIb) (Arhab et al., 2020). Despite the high variability of dII in the HCV IRES, its apical hairpin loop, internal loop E motif and basal internal loops are conserved among HCV, and closely related to those of HCV-like IRESs (Lukavsky, 2009). Notably, dIV in HCV is not unique to it. This special structure is also found in some picornaviral genera (e.g., *Colbovirus*, *Megrivirus*, *Sapelovirus*, *Crohivirus*, *Pasivirus*, and *Limnipivirus*) containing the type IV IRES. The dIV in these genera shows the lower stability than the homologous region in HCV (Asnani et al., 2015).

Collectively, the type IV IRES contains a highly conserved core region, which is crucial for the translation initiation. Surrounding this core, other regions, e.g., dII, vary significantly among IRESs from different species. This combination of structural conservation and variability may be related to the function and regulation of type IV IRES.

## 5 Functions of type IV IRES in picornaviruses

The heterogeneity of type IV IRES structures exemplifies biological variety, and equips viruses with adaptive mechanisms to thrive in various host environments (Martinez-Salas et al., 2017). Each IRES configuration is likely tailored to particular environmental and biological demands, facilitating viral proliferation and transmission across diverse hosts. Cellular proteins binding to viral IRES are critical for orchestrating the life cycle of viruses. For example, certain cellular proteins can enhance the activity of IRES, while others may suppress its function (Liu et al., 2020; Embarc-Buh et al., 2021; Lopez-Ulloa et al., 2022; Marques et al., 2022). During this process, the secondary and tertiary structures of IRES constitute the foundation for the interaction with various cellular factors. Perturbation of specific conserved motifs may compromise IRES structural integrity, potentially impeding viral replication.

The flexibility of secondary structure and the uniqueness of primary sequence within dIII contribute to its role as the most active region in RNA–protein interactions (Barría et al., 2009; El Awady et al., 2009; Lukavsky, 2009; Johnson et al., 2017). The dIIIa, dIIIb, and dIIIc of HCV are primarily involved in the binding of eIF3 and the 40S subunit (Kieft et al., 2001; Rijnbrand et al., 2004). Elements equivalent to HCV dIIIa and dIIIc are less conserved in picornaviral IRESs, possibly implying that these domains are less critical for their functional activities. Nevertheless, the possibility that these two domains still have other functions in picornaviral IRESs cannot be wholly dismissed, and these functions may be achieved by the evolution of additional sequences (Chard et al., 2006b). The interaction between dIIIb and eIF3 affects a key process of active ribosomal assembly, during which the binding of eIF3 to dIIIb will exert effects on both eIF3 and eIF2 stabilities in the formation of preinitiation complexes (Easton et al., 2009). Deletion mutations at this site reduce initiator tRNA deposition, leading to further compromise of the 80S complex assembly (Ji et al., 2004). Although the “8”-like structure is present in the apical region of dIIIb in certain instances, a comprehensive research on its precise mechanism of action remains to be conducted further. A limited number of studies (Boros et al., 2014b) have documented the impact of deletions or mutations in this structure on IRES functionality. However, given its prevalent occurrence in avian picornaviruses, the structure can hold unique significance.

The GGG motif in the dIIId loop is a major determinant of ribosome binding dIIId (Jubin et al., 2000; Friis et al., 2012; Niepmann et al., 2018), capable of base-pairing with CCC nucleotides in the apical loop of ES7 of 18S ribosomal RNAs (Hashem et al., 2013; Lattimer et al., 2019). The dIIId apical loop contains a GG motif in the genus *Pemapivirus*, indicating that this dinucleotide sequence may be adequate for ribosomal binding (Arhab et al., 2020). Mutations in this sequence can reduce the binding affinity of the IRES for the 40S subunit, thereby hindering the assembly of the 48S complex (Ji et al., 2004; de Breyne et al., 2008; Willcocks et al., 2011; Pan et al., 2012). Certain viruses possess two dIIId: dIIId1 and dIIId2. The latter exhibits the variability in function across various viruses. For instance, dIIId2 in senecavirus A (SVA), albeit unessential for IRES-mediated translation as evidenced by its non-impact on eIF3 and 40S recruitment, 48S complexes and 80S ribosomes assembly, plays a crucial role in viral infectivity (Willcocks et al., 2011). The absence of dIIId2 would interfere with the assembly of 80S ribosome in both BDV- and CSFV-infected cells, consequently impeding the translation initiation (Willcocks et al., 2017).

The GA[U/C]A tetraloop observed in the dIIIe region of HCV-like IRES diverges from the standard GNRA tetraloop that is characteristic of type I and II IRES elements (López de Quinto and Martínez-Salas, 1997; Psaridi et al., 1999; Robertson et al., 1999; Asnani et al., 2016; Mailliot and Martin, 2018). Despite minor variations in the tetraloop bases across IRES types, their nucleotide sequences are all essential for IRES function. Nucleotide insertions, mutations or deletions in this motif may lead to IRES inactivation, thereby affecting the conformation and stability of IRES by modulating the interaction of RNA–RNA tertiary structures (Bhattacharyya and Das, 2006; Fernandez-Miragall et al., 2006; Bakhshesh et al., 2008; Pan et al., 2012). For example, the apical part of dIII would be reorganized in the FMDV IRES after a single nucleotide substitution in the GNRA motif to GUAG, or its substitution by a UNCG motif (Fernandez-Miragall and Martinez-Salas, 2003). Change in each of the four nucleotide positions in the GAUA loop of HCV IRES severely impairs the IRES activity (Psaridi et al., 1999), and this hairpin loop may enhance the interaction between the IIId and the 40S subunit (Lukavsky et al., 2000; Angulo et al., 2016).

Type IV IRES features a single PK structure, in contrast to three PKs in dicistroviruses (Abaeva et al., 2023). The structure is formed via base pairing between loop nucleotides of IIIf and other single-stranded regions, showing two base-paired stems, stem I and II (Liu et al., 2021a). The IRES activity is dependent both on the primary sequence and on the secondary structure in both stem regions. Any mutation, if responsible for disruption of the secondary structure of stem region, mutations on both sides of the stem sequence without disturbing the overall conformation, or insertion (or deletion) mutations in the stem region, would invariably compromise the IRES function (Chard et al., 2006b; Berry et al., 2010; Willcocks et al., 2011; Pan et al., 2012; Wang et al., 2021; Liu et al., 2021b). Furthermore, sequence alterations within the PK region of SVA stem II can potentially exert an impact on virion assembly (Liu et al., 2021a). The function of the PK is contingent upon its intact structure. When the PK participates in the binding of the 40S subunit, its primary role is associated with positioning the start codon to the ribosomal P site (Kieft et al., 2001; Chard et al., 2006b; Lukavsky, 2009). An optimal distance is also maintained between the PK and the start codon. Either insertion or deletion of nucleotides in this sequence would interfere with viral replication to some extent, possibly attributed to deleterious effects on the AUG reaching the P site (Berry et al., 2010; Liu et al., 2021c).

Ribosomal translocation is a crucial stage in the protein synthesis, requiring the mRNA template to move so that new codons are positioned within the A site for decoding (Zhou et al., 2014; Milicevic et al., 2024). The conformational shift induced by HCV dII in the 40S subunit affects the translocation process (Brown et al., 2022). Despite the spatial separation between dII and dIII, the asymmetrical interior loop in dIIa facilitates the formation of bent dII conformation (Lukavsky et al., 2003). This bending permits the apical region of dIIb to spatially contact the 40S ribosomal subunit bound to dIII, not only facilitating the adoption of a specific HCV RNA configuration in the decoding groove, but also potentially assisting in the proper selection of start codon (Lukavsky et al., 2003; Filbin and Kieft, 2011; Johnson et al., 2017; Brown et al., 2022).

The hairpin loop and loop E motif in dIIb can promote both eIF5-induced GTP hydrolysis and eIF2/GDP release from the initiation complex (Locker et al., 2007; Martinez-Salas et al., 2008; Barría et al., 2009). Both deletion and mutation of the dII region would lead to a significant reduction in the IRES function (Friis et al., 2012; Filbin et al., 2013). The majority of functional studies are currently involved in the dII of HCV IRES. The dII in the type IV IRES shows considerable variation among picornaviruses, some of which contain neither a loop E motif nor an asymmetric inner loop. In certain viruses, their dII regions simply show a stem-loop structure formed via a 21-nt-long motif (Asnani et al., 2015). This simplified dII may exert functions distinct from those of HCV through synergistic actions with other IRES domains, or interaction with specific host factors, reflecting the evolutionary plasticity of viral IRES in both structure and function.

To summarize, IRES dIII primarily engages in the binding of eIF3 and 40S ribosomal subunits, and in the precise localization of the start codon. The dII is mainly involved in 80S ribosome assembly, and in the following initiation steps. The attachment of 40S ribosomal subunit or eIF3 to IRES is not confined to an individual dIII subdomain. Rather, the higher-order structures formed between multiple IRES domains, along with their exposed conserved sequences and structural motifs, collectively provide multiple regulatory interfaces for recruitment and precise localization of these molecules. Therefore, maintaining the structural integrity of the IRES is functionally crucial for viral replication. Any mutation, which disrupts the IRES structure or alters its conserved sequences, will lead to IRES inactivation, thereby affecting the translation process of the viral proteins.

## 6 Conclusion

The first reports concerning IRES elements unequivocally demonstrated the cap-independent initiation of translation in PV and EMCV genomes. There has been substantial progress in recent 20 years in uncovering structures and functions of picornaviral IRESs. The type IV IRES can fold into a compact tertiary structure, making its own key elements interact with the small ribosomal subunit through a cluster of specific ribosomal proteins for triggering the translation of viral polyprotein. Unfortunately, most valuable reports associated with the type IV IRES are based on the HCV, rather than on picornaviruses. There is still much to be learned about the interaction of type IV IRES with host factors or even other RNA elements. Nowadays, characterizing its high-order organization of RNA structure, in conjunction with the recognition of IRES–protein interaction network, is the greatest challenge to unveil how these specialized RNA structures function during viral replication.

## Data availability statement

The original contributions presented in the study are included in the article/Supplementary material, further inquiries can be directed to the corresponding authors.

## Author contributions

YL: Writing – original draft. LZ: Writing – original draft. LW: Writing – original draft. JL: Software, Writing – original draft. YZ: Software, Writing – review & editing. FL: Conceptualization, Writing – review & editing. QW: Writing – original draft, Writing – review & editing.

## Supplementary material

Supplementary Figure S1 Representative structures of type IV IRESs potentially forming in picornaviral genomes. The conserved motifs in DII and DIII are indicated by blue shades. The start codon is indicated by a gray shade.
