# Supplementary figures and images for: Structure and function of type IV IRES in picornaviruses: a systematic review
Source: Front Microbiol. 2024 May 24;15:1415698. doi: 10.3389/fmicb.2024.1415698 (PMC11157119; doi:10.3389/fmicb.2024.1415698)

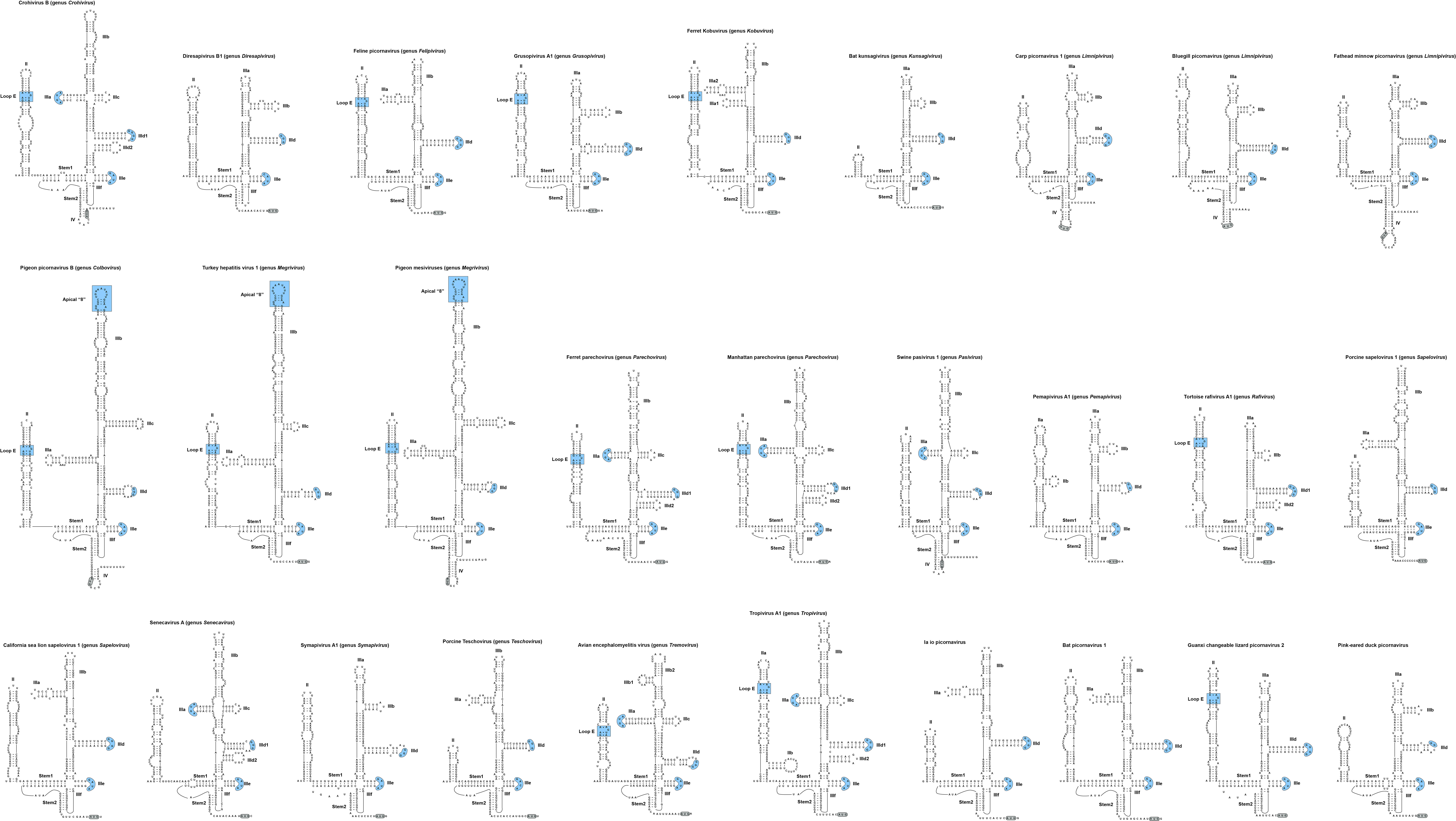

Supplement: Supplementary Figure S1 — Representative structures of type IV IRESs potentially forming in picornaviral genomes. The conserved motifs in DII and DIII are indicated by blue shades. The start codon is indicated by a gray shade. [file Image_1.JPEG]
